# The influence of extracorporeal membrane oxygenation therapy on intestinal mucosal barrier in a porcine model for post-traumatic acute respiratory distress syndrome

**DOI:** 10.1186/s13019-015-0211-3

**Published:** 2015-02-15

**Authors:** Ling Ni, Qiyi Chen, Ke Zhu, Jialiang Shi, Juanhong Shen, Jianfeng Gong, Tao gao, Wenkui Yu, Jieshou Li, Ning Li

**Affiliations:** 1 Research Institute of General Surgery, Jinling Hospital, Medical school of Nanjing University, 305 East Zhongshan Road, Nanjing, 210002 Jiangsu Province China; 2 Catheter Room of Yongcheng People’s Hospital of Henan Province, Yongcheng, Henan Province China

**Keywords:** Extra-corporeal membrane oxygenation, Immune inflammatory response, Intestinal mucosal, Adult respiratory distress syndrome

## Abstract

**Background:**

It is unclear at present whether extracorporeal membrane oxygenation (ECMO) therapy can improve intestinal mucous barrier function through increased perfusion. The present study establishes an animal model for post-traumatic acute respiratory distress syndrome (ARDS) and evaluates the effect of v-vECMO treatment on the intestinal mucosal barrier.

**Method:**

Pulmonary contusion combined with ischemia-reperfusion injury was induced in 30 piglets. The animals were randomly divided into control, model, and ECMO groups. Serum I-FABP, d-lactate, and endotoxin were measured over a 24-h period. The jejunum and colon were collected post-mortem and evaluated histopathologically. The tissue was also examined using electron microscopy, and intestinal tight junction proteins (ZO-1 and occludin) were measured after 24 h of ECMO therapy. Mortality rate and cause of death were also recorded.

**Results:**

The serum markers evaluating the intestinal mucosal barrier deteriorated in the model group compared to the control group (p < 0.05). At 2 h, serum I-FABP, d-lactate, and endotoxin were significantly increased in the ECMO group compared to the model group (p < 0.05). At 12 h, I-FABP and d-lactate in the ECMO group dropped to model group levels. Serum d-lactate was slightly lower in the ECMO group (p > 0.05) and serum I-FABP was significantly lower than in the model group (p < 0.05) at 24 h. Similarly, serum endotoxin was slightly lower in the ECMO group than in the model group (p > 0.05) at 24 h. After 24 h of ECMO therapy, the occludin and ZO-1 protein concentrations in jejunum and colon mucosa increased moderately compared to that in the model group (p < 0.05). Morphologic structure of the jejunum and colon did not improved significantly after ECMO therapy. Finally, we observed that ECMO therapy moderately decreased mortality (25% vs. 50%).

**Conclusion:**

Intestinal mucosal barrier continued to deteriorate in the model group. Although early ECMO therapy aggravates intestinal mucosal injury, the damage gradually improves later during therapy. The results show that ECMO therapy has a protective effect on the intestinal mucosal barrier in the later treatment stage.

## Background

Extracorporeal membrane oxygenation (ECMO) is an effective treatment for patients suffering from severe acute respiratory distress syndrome (ARDS). When cardiovascular and pulmonary function is impaired, ECMO ensures adequate gas exchange and tissue perfusion using an artificial gas-exchange membrane and blood pumps. It quickly stabilizes homeostasis, reduces mechanical ventilation parameters, and offers opportunities for the diagnosis and treatment of the primary disease [1,2]. ECMO also provides an opportunity for the lung to rest, thus avoiding iatrogenic ventilator-associated lung injury [3-5]. The successful use of ECMO in the 2009 H1N1 influenza pandemic brought ECMO into the worldwide spotlight, and VV-ECMO is being popular [6,7].

The gastrointestinal tract is a driver of multi-organ dysfunction syndrome (MODS) in critically ill patients [8]. The intestines receive 20% of cardiac output and 20–35% of systemic oxygen delivery under rest conditions. Unfortunately, blood flow and oxygen supply to the gastrointestinal tract is reduced under critical conditions to maintain brain and heart perfusion. Some studies suggest that the pH decreases and causes intestinal mucosal injury when the intestinal oxygen supply decreases to 50–60% [9,10]. The gastrointestinal bacterial population is an estimated at 10^14^; when the mucosal barrier is destroyed, these bacteria translocate across the intestines into the vasculature, resulting in severe infection and MODS [8]. In addition, hypoxia disrupts the bacteria themselves, leading to intestinal flora disorder [11]. Therefore, quick restoration of intestinal perfusion is important for the prognosis of critically ill patients. In patients diagnosed with severe ARDS, ECMO significantly improves oxygen supply, and Vein-Artery ECMO partially replaces heart function by increasing blood pressure. Theoretically, ECMO therapy could improve intestinal mucosal barrier function by increasing oxygen and blood perfusion.

ECMO is a relatively new technology; therefore, further research is needed to ascertain its clinical utility. Our previous studies revealed that ECMO therapy may injure the heart [12,13], kidney [14], and brain [15]. Other recent case reports show that ECMO therapy can increase intra-abdominal pressure [16,17]. Notably, one animal study reported that ECMO therapy may destroy the intestinal mucosal barrier and increase the incidence of bacterial translocation [18]. To date, only few case reports have discussed clinical application of ECMO, and no relevant randomized controlled trials currently exist. Critically ill patients already have an increased likelihood of elevated intra-abdominal pressure and resulting abdominal compartment syndrome. Therefore, the relationship between ECMO, abdominal hypertension, and abdominal compartment syndrome warrants investigation.

In a previous animal study, the effects of ECMO therapy on the intestinal mucosa were not evaluated under hypoxic or ARDS conditions (ischemia-reperfusion animal models). Mean treatment time for ECMO was only 8 h, but in clinical practice, ECMO may need to be administered for a far longer period, making the study inadequate under present therapeutic guidelines. Our previous studies of ECMO were performed in normal healthy animals. Other studies suggest potential intestinal mucosal damage during ECMO treatment, but these effects were likely related to the underlying disease rather than being a therapeutic complication [19,20].

Therefore, the present study, using an ARDS animal model, investigates whether ECMO therapy administered under ARDS conditions improves intestinal mucosal barrier by correcting oxygen delivery and carbon dioxide removal or instead injures the mucosa.

## Methods

### Animal preparation and group distribution

This study was approved by the Animal Care Committee of Jingling Hospital. Thirty piglets of either sex weighing 30 ± 5 kg were used. The animals received ketamine (20 mg/kg IM), diazepam (8 mg/kg IM), and atropine (0.1 mg/kg IM) prior to anesthetic induction. Then, ketamine (10–20 mg/kg/h IV) and diazepam (8 mg/kg/h IV) were administered to maintain anesthesia. Once anesthetized, each piglet was secured to the surgical table insulated with a blanket to prevent hypothermia. The piglets were randomly assigned into either the control group (n = 6), model group (n = 12), or ECMO group (n =12).

### Catheterization and coagulation monitoring

Once the pigs were anesthetized, tracheotomy was performed and a 6.0-mm internal diameter tracheal tube was placed immediately. Then, a 16-gauge venous catheter was placed into the left internal jugular vein and Lactated Ringer’s solution was administered at 3 mL/kg/h initially. The rate was increased as needed to maintain the mean arterial pressure above 60 mmHg. A 16-gauge catheter was inserted into the right femoral artery to monitor blood pressure. Heparin (150 U/kg IV) was administered and a 14-French Biomedicus venous drainage cannula (Medtronic Perfusion Systems, Minneapolis, MN) was inserted into the right femoral vein, followed by a 14-French Biomedicus arterial cannula (Medtronic Perfusion Systems) into the internal jugular vein for intravenous infusion. Heparin was infused to maintain the activated clotting time at 180–220 s. Correct cannula placement was confirmed using ultrasonography.

### Establishing post-traumatic ARDS model by pulmonary contusion and ischemia-reperfusion

After the catheters were successfully inserted, subjects in the model and ECMO groups were positioned laterally. Adjustable (height and width) smooth cylindrical hollow columns, measuring 100-cm high and 20 cm in diameter, were fixed to the vertical surfaces of ribs 6–9. A second smooth metal cylindrical column (weight 0.45 kg/kg of pig) was suspended vertically and centrally above the attached columns. The metal column was positioned parallel to the hollow column edge using a rope. The rope was then cut, which allowed the metal column to free fall and hit the chest wall of the pig, establishing pulmonary contusion. The procedure was then repeated on the other lateral side.

After a lung contusion was produced, hemorrhage was induced through the jugular venous catheter in order to decrease the mean arterial pressure to 40 ± 5 mmHg within 10 min; mean arterial pressure was maintained at this level for 2 h to establish ischemia. Afterwards, in the first hour, Lactated Ringer’s solution was administered through the jugular vein at 3 times the blood loss volume, and 30% of the hemorrhaged blood was administered through the ear vein. At the second hour, Lactated Ringer’s solution equal in volume to the hemorrhagic loss was administered. At the third hour, reperfusion was established, and Lactated Ringer’s solution was administered, guided by PICCO_2_. The fluid rate was adjusted as needed to maintain the mean arterial pressure at 90 mmHg.

### Experimental protocol

After baseline measurements, animals were randomly divided into three groups: the control group (n = 6), the model group (n = 12), and the ECMO group (n = 12). Because of limitations of animal body weight and blood volume, it is difficult to increase the oxygen supply by increasing the flow. In our preliminary experiments results show it is most appropriate at 50 ml/mim/kg for ECMO circulation. So have no sub groups were divided according to oxygen concentration in EMO group. At hour 0, the vascular venous cannula was occluded in the control and model groups, and the VV-ECMO was established in the ECMO group. To measure potential oxidative stress injury associated with the ECMO procedure, presurgical venous blood samples were collected from the ear vein. Because the ECMO procedure can be completed within 1 hour, the initial baseline was set to −1 hour.

### VV-ECMO procedure

The ECMO circuit (Quadrox PLS, Maquet, Germany) was primed with 500 mL of Voluven and 200–300 mL of Lactated Ringer’s solution. The venovenous-ECMO system comprises a centrifugal pump (ROTAFLOW Console, Maquet, Germany) and a heat exchange (Heater-Cooler Unit HCU 30, Maquet, Germany) maintaining temperature of 37°C. Sweep gas was 100% oxygen at a flow rate equal to the blood flow rate (1:1). Blood within the circuit was drained from the right femoral vein and infused into the right internal jugular vein at 50 mL/kg/min.

### Mechanical ventilation strategy

Once ARDS was established, mechanical ventilation was begun in a volume-controlled mode with a 0.50 FIO^2^ and a 5 mmHg positive end-expiratory pressure. Tidal volume was 8 mL/kg and respiratory rate was 15 breaths/min.

### Sample collection

During ECMO treatment, blood samples were collected from the right femoral artery at −1, 0, 2, 6, 12, and 24 h. Samples were centrifuged at 2500 rpm for 15 min at 4°C, and the plasma was stored at −70°C for measurement of serum d-lactate, IFBAP, and endotoxin.

At the end of the 24-hour ECMO treatment period, all animals were euthanized with intravenous potassium chloride (40 mL, 0.1 g/mL). Jejunum and colon samples, including mucosal layers, were collected for histopathology, electron microscopy, and intestinal mucosa protein evaluation.

## Examination of sample

### Serum intestinal fatty acid-binding protein (I-FABP) measurement by ELISA

Serum I-FABP was measured at baseline, 0, 2, 6, 12, and 24 h before and during ECMO by spectrophotography using commercial analysis kits (Sunredbio, USA).

### Measurement of D-lactate in serum by spectrophotography

Serum d-lactate was measured at baseline, 0, 2, 6, 12, and 24 h before and during ECMO by spectrophotography using commercial analysis kits (Abnova, USA).

### Serum endotoxin measurement by limulus amebocyte lysate test

Serum endotoxin was measured using a limulus amebocyte lysate test at baseline, 0, 2, 6, 12, and 24 h before and during ECMO therapy by chromogenic end point assay (Limulus Amebocyte Lysate Kit; LAL Test Company, Xiamen, China).

### Measurement of occludin and ZO-1 in intestinal mucosa by western blot

Western blot analyses were performed to measure occludin and ZO-1 protein expression. Segments of distal jejunum and colon were excised, and the mucosa was isolated by scraping. Proteins were extracted using the RIPA buffer for analysis and protein concentration determined by bicinchoninic acid assay (Pierce Biochemical). Equal amounts of each extract were electrophoresed in 6% SDS–PAGE and electrotransferred onto PVDF membranes (Bio-Rad). The membrane was blocked for 90 min at room temperature in 3% bovine serum albumin and 0.1% Tween-20 in Tris-buffered saline (TBS), followed by overnight incubation with primary antibodies diluted in 1% bovine serum albumin in TBS at 4°C. Membranes were washed three times in TBS (containing 0.1% Tween-20) and then incubated with the secondary antibodies for 90 min at room temperature. The membrane was developed by incubating it for 5 min in Super Signal Reagent (Pierce). Band intensities were quantified by densitometry and expressed as the mean area density using Quantify One software (Bio-Rad), version 4.1.1. For total protein measurement, mean area density was calculated relative to GAPDH expression.

### Histopathology

Segments of distal jejunum and colon were excised, transferred into 10% phosphate-buffered saline (PBS) and buffered formalin, and then embedded in paraffin. Sections were cut to 5-mm, mounted on glass slides, and stained with hematoxylin and eosin (H&E). Images were obtained using a Zeiss Image A1 light microscope at 100× magnification and AxioVision V4.5 software. Three pathologists, blinded to the slide identities, evaluated each slide. Histopathology was graded semiquantitatively using the histologic injury scale described by Chiu et al. [21].

### Transmission electron microscopy of tight junctions

Sections of the jejunum and colon (2-mm thick) were washed and fixed with 4% glutaraldehyde for 2 h and post-fixed with 1% osmium tetroxide. Tissues were embedded in Epon 812 and thinly sectioned. Thin sections were cut and stained with uranyl acetate and lead citrate and examined with an H-600 (Hitachi, Japan) transmission electron microscope operated at 75 kV.

## Statistical analysis

Data were analyzed using SPSS 17.0. Mortality comparison between the model and ECMO groups was performed using χ^2^. Other differences in data were determined using one-way ANOVA (multiple group pairwise comparison by LSD test). The p values < 0.05 were considered statistically significant. Results are presented as mean ± SD.

## Results

### Mortality and cause of death

4 animals died within 2 h after injury from severe pneumothorax and bleeding ( chest, heart and liver rupture), as they did not die of mucosal barrier injury they were discarded from the analysis. In the model group, 6 of 12 pigs (50%) died from multiple causes, including refractory hypoxemia (4), hypothermia and coagulation disorders (1), and congestive heart failure (1). During ECMO therapy, 3 of 12 pigs (25%) died from two causes: congestive heart failure (2) and coagulation disorders and acidosis (1).

### ECMO therapy attenuates I-FABP proteins concentrations in serum for post-traumatic ARDS porcine model (Figure 1)

**Figure 1:**
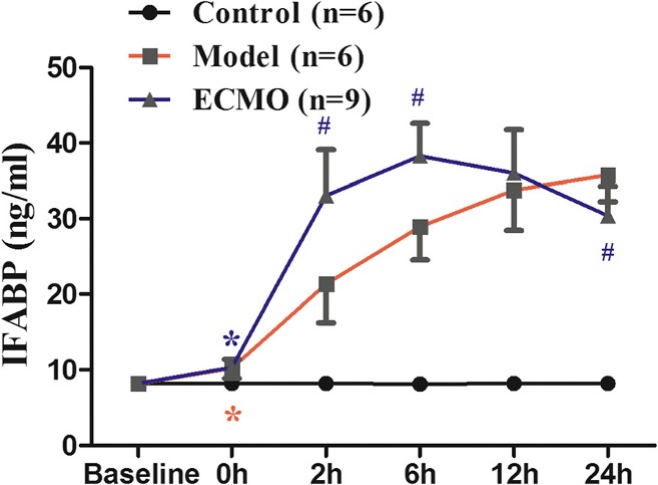
**Effect of ECMO therapy on IFABP in serum in different time points.** Value are ex pressed as mean+/− SD of each group. Compared with control group, *p < 0.05, compared with Molde group, #p < 0.05.

I-FABP concentration remained normal in the control group. After establishing post-traumatic ARDS (0 h), the I-FABP was significantly higher (p < 0.05) in the model and ECMO groups than in the control group. I-FABP increased gradually in both the model and ECMO groups; however, I-FABP in the ECMO group was significantly higher than in the model group 2 h later, a trend lasting until 6 h after post-traumatic ARDS (p < 0.05). The ECMO I-FABP then decreased gradually, equaling the model group at 12 h (p > 0.05) and declining below the model group level at 24 h (p < 0.05).

### ECMO therapy attenuates D-lactate concentrations in serum for post-traumatic ARDS porcine model (Figure 2)

**Figure 2:**
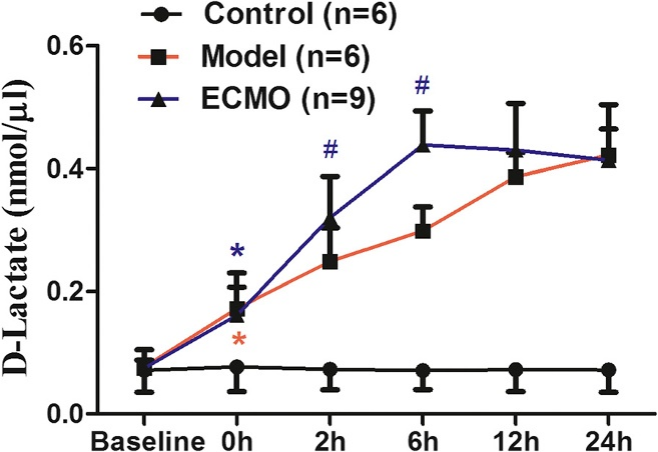
**Effect of ECMO therapy on D-Lacate in serum in different time points.** Value are expressed as mean+/− SD of each group. Compared with control group, *p < 0.05, compared with Model group, #p < 0.05.

d-lactate concentration remained normal in the control group. After establishing post-traumatic ARDS (hour 0), the serum d-lactate significantly increased (p < 0.05) in the model and ECMO groups compared to the control group. Serum d-lactate increased gradually in both the model and ECMO groups, but the increase was significantly higher in the ECMO group at 2 h and until 6 h (p < 0.05) after ARDS was established. Serum d-lactate then decreased gradually in the ECMO group, equaling the model group at 12 h (p > 0.05) and decreasing slightly below the model group level at 24 h (p > 0.05).

### ECMO therapy attenuates Endotoxin concentrations in plasma for post-traumatic ARDS porcine model (Figure 3)

**Figure 3:**
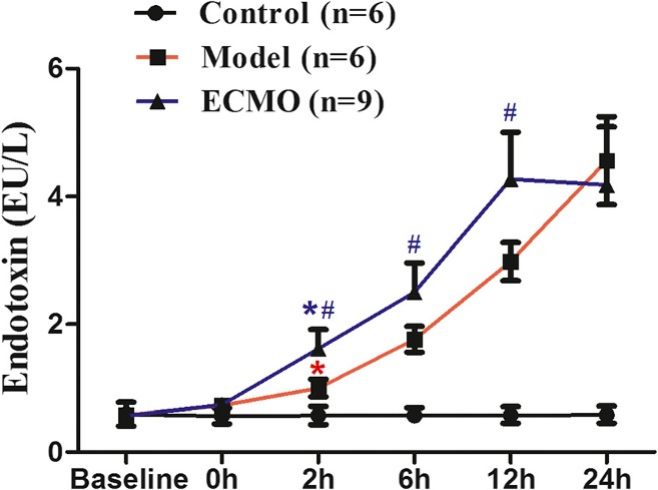
**Effect of ECMO therapy on endotoxin in serum in different time points.** Value are expressed as mean+/− SD of each group. compared with control group, *p < 0.05, compared with Model group, #p < 0.05.

Serum endotoxin remained normal in the control group. Two hours after establishing post-traumatic ARDS, serum endotoxin in the model and ECMO groups were significantly increased compared to the control group (p < 0.05). Serum endotoxin increased gradually in both the model and ECMO groups, but the increase was higher in the ECMO group at 2 h and until 12 h than observed in the model group (p < 0.05). Serum endotoxin then decreased gradually in the ECMO group and was slightly lower than the model group level at 24 h (p > 0.05).

### ECMO therapy enhances tight junction proteins concentrations in jejunum and colon for post-traumatic ARDS porcine model (Figures 4, 5, 6 and 7)

**Figure 4:**
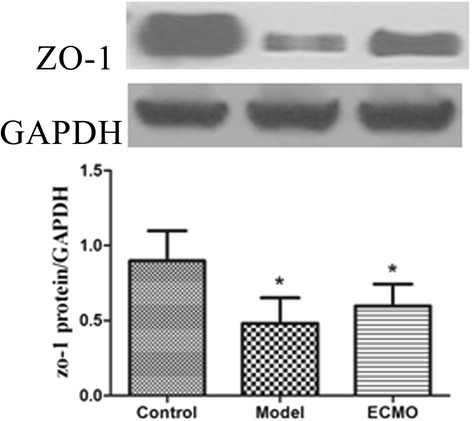
**Tight junction proteins ZO-1 change in jejunum.** The protein analysed by western blot with protein levels quantified by densitometry and normalized to GAPDH. compared with Control group, *p < 0.05.

**Figure 5:**
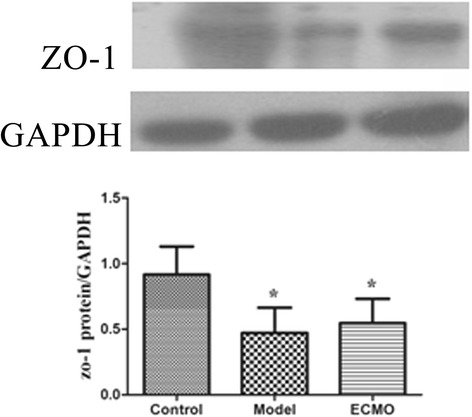
**Tight junction proteins ZO-1 change in colon.** The protein analysed by western blot with protein levels quantified by densitometry and normalized to GAPDH. compared with Control group, *p < 0.05.

**Figure 6:**
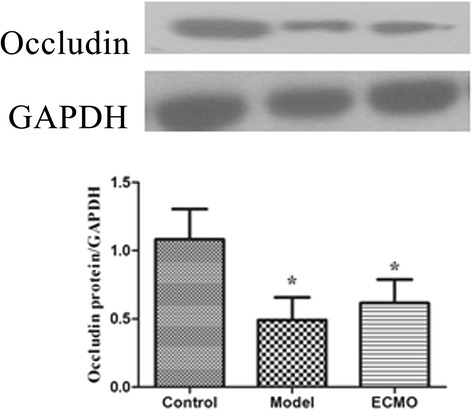
**Tight junction proteins Occludin change in jejunum.** The protein analysed by western blot with protein levels quantified by densitometry and normalized to GAPDH. compared with Control group, *p < 0.05.

**Figure 7:**
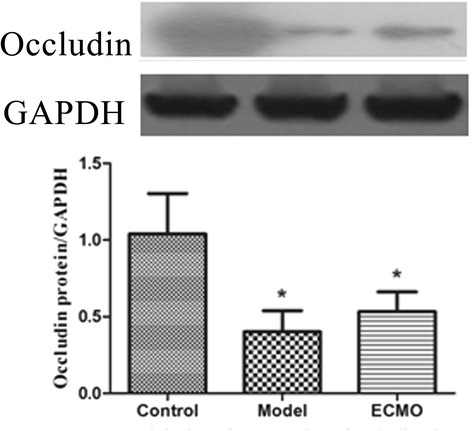
**Tight junction proteins Occludin change in colon.** The protein analysed by western blot with protein levels quantified by densitometry and normalized to GAPDH. compared with Control group, *p < 0.05.

Compared to the control group, the occludin and ZO-1 protein concentrations in jejunum and colon mucosa decreased significantly in the model group (p < 0.05). After 24 h, occludin and ZO-1 concentrations moderately increased in the ECMO group compared to the model group, but this difference was not statistically significant.

### ECMO therapy attenuates pathological injury in jejunum and colon for post-traumatic ARDS porcine model (Figure 8)

**Figure 8:**
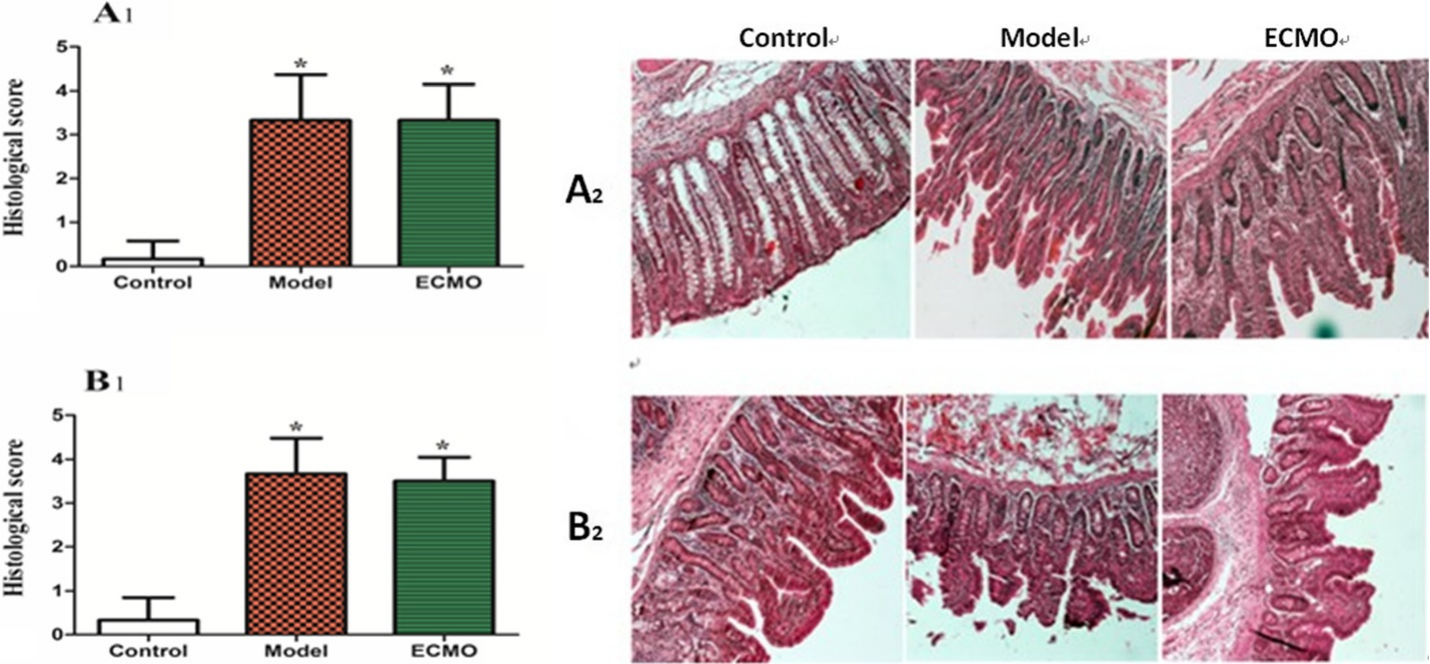
**Histopathologic evaluation (×40) of jejunum and colon by HE staining in ARDS model pigs.** Chiu’s scores in jejnum **(A1)** and colon **(B1)** experresed as mean ± SD of the controlgroup, model group, and ECMO group, with *p < 0.05, **A2** and **B2** represed pathologic changes in jejunum and colon, respectively.

In the control group, subjects had integrated villi and a compact epithelium. The jejunum and colon in both the model and ECMO groups had a disordered mucosal structure, marked atrophy, and blunted villi. In addition, the brush border was discontinuous and the epithelium disarrayed. Chiu’s score for the model group was higher than that for the control group (p < 0.05). At 2 h, the ECMO group intestinal injury score was not statistically different from the model group score (p > 0.05).

### ECMO therapy attenuates ultra-structure injury in jejunum and colon for post-traumatic ARDS porcine model (Figure 9)

**Figure 9:**
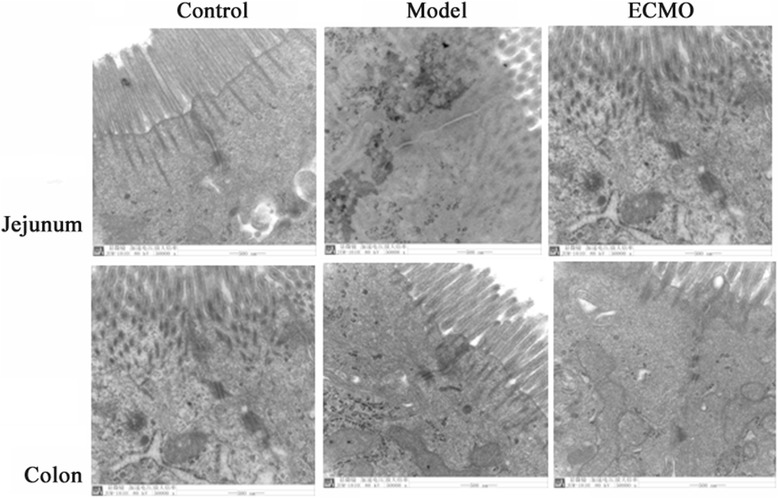
**Ultrastructural change in jejunum and colon using electron mocroscopy.**

Electron microscopy (×50000) showed no significant abnormalities in structural integrity of tight junctions and microvilli in the control group. In the model group, epithelial tight junctions in the jejunum and colon were loosened and the gap widened; there was also microvilli loss, and desmosomes density decreased. After 24 h of ECMO therapy, the intestinal mucosal injury was not affected.

## Discussion

The present study establishes an post-traumatic ARDS animal model by inducing chest contusion combined with ischemia-reperfusion injury. Our preliminary experiment results showed that the PaO2/FIO2 ratio decreased to 206.35 ± 18.97, and computer tomography (CT) examination showed bilateral diffuse caudal pulmonary dense radio-opacity (data not shown) after traumatizing the thorax, which confirms that the model was consistent with ARDS conditions. ARDS can result in systemic organ hypoxia, and systemic oxygen consumption increases notably under ARDS conditions [22]. Unfortunately, blood flow and oxygen supply to the gastrointestinal tract decreases under critical conditions to maintain perfusion to vital organs such as brain and heart. The gastrointestinal tract is a driver of multi-organ dysfunction syndrome (MODS) in critically ill patients. When exposed to hypoxic conditions, the intestinal mucosal barrier is destroyed and bacteria in the gastrointestinal tract translocate into the blood, resulting in severe infection and progressing to MODS [8]. In a related study, mortality reached 50%, and 66.77% of subjects died from hypoxia under post-traumatic ARDS conditions. Meanwhile, intestinal mucosa was continually injured under the hypoxic condition. Therefore, improving intestinal mucosal oxygen supply may be an important means to prevent intestinal mucosal injury and MODS in critically ill patients.

ECMO therapy could improve the prognosis of critical patients by increasing intestinal tissue oxygen delivery, which protects the gastrointestinal mucosa barrier. Thus, we investigated the effect of ECMO treatment on jejunum and colon mucosa in ARDS model pigs. I-FABP is a 15KD protein and exists only within the intestinal epithelium, primarily in the small intestinal microvilli tip [23]. It is an early and sensitive indicator of intestinal damage. d-lactate is also a sensitive indicator of intestinal permeability. However, after 2 h of ECMO therapy, I-FABP, d-lactate, and endotoxin levels were actually higher in the ECMO group than those observed in model group subjects. Serum I-FABP and d-lactate levels remained consistently higher in the ECMO group than those in model group subjects until 6 h after initiating ECMO therapy; serum endotoxin remained higher in the ECMO group than in the model group until 12 h. These results suggest that ECMO treatment does not improve the intestinal mucosal barrier, but may actually severely damage the tissue at 2–12 h post-injury.

An animal study found that ECMO therapy can increase serum I-FABP and d-lactate 1 hour after perfusion injury until a peak at 8 h, which is accompanied by the destruction of intestinal tight junctions and bacterial translocation [16]. The intestinal damage was suspected to be related to an immune inflammatory response during early ECMO therapy [16]. Our previous research suggested that immune mediated inflammation occurs 2 h after initiating ECMO therapy. After 24 h of ECMO therapy, multiple organs, such as the kidney, brain, and myocardium, upregulated expression of inflammatory cytokine [12-14]. In addition, a few rare clinical case reports suggest that ECMO therapy may increase serum endotoxin [24]. In our clinical experience, bacteremia may be noted in approximately 50% of patients receiving ECMO therapy, but after discontinuing ECMO and extubating the patient, the bacteremia disappears within 24 h, and we suspected that the bacteremia was associated with a catheter infection.

Despite these findings, the previous studies had several limitations. Notably, the influence of ECMO therapy on the intestinal mucosa barrier still needs to be observed for a longer period mimicking typical clinical conditions. In the present study, we observed the effect of ECMO treatment on the intestinal mucosa for an extended period. After 12 h of ECMO therapy, we found that d-lactate and I-FABP decreased significantly to model group levels. After 24 h of ECMO therapy, the serum I-FABP and endotoxin were lower than those observed in the model group. These findings suggest that early intestinal mucosal damage was ameliorated gradually and continued to improve during an extended period of ECMO therapy. In contrast, the model group showed continued mucosal deterioration. We observed that ECMO therapy can decrease the mortality of post-traumatic ARDS pigs (ECMO group: 25%; model group: 50%) and potentially eliminate hypoxia-induced mortality. We speculate that a reduction in intestinal tissue hypoxia associated with ECMO therapy may offset the initial immune inflammatory response during therapy. Long-term intestinal tissue hypoxia causes more serious adverse effects than the immune inflammatory response and tissue edema resulting from other factors.

After 24 h of ECMO therapy, all animals were euthanized to evaluate morphology and tight junction proteins ZO-1 and occludin in the jejunum and colon. In the Model group, the concentrations of occludin and ZO-1 were decreased significantly, and the morphology was significantly disordered. After 24 h of ECMO therapy, there was no morphological evidence of injury to the intestinal mucosa in the ECMO group. The occludin and ZO-1 protein concentrations were increased moderately in the ECMO group, but there was no statistical difference between the model and ECMO groups. The hematologic results suggest significant improvement in injured intestinal mucosa, but the intestinal morphologic structures did not show these same improvements after 24 h of ECMO therapy. This discrepancy may reflect a gradual recovery of the intestinal mucosa, which may require a longer time to detect histologically.

### Limitations

This study uses animal models to mimic ARDS, but the methodology is not widespread globally. Furthermore, the lack of morphologic change between the model and ECMO groups may signal a need for observation over a longer time.

## Conclusion

Our study shows that ECMO therapy aggravates intestinal mucosa injury early during therapy, but the injury declines gradually. Furthermore, ECMO therapy has a protective effect on the intestinal mucosal barrier in the later treatment stage. Finally, ECMO therapy decreases post-traumatic ARDS associated mortality, and may eliminate hypoxia-associated mortality. The lack of morphologic change in the intestinal mucosa following ECMO therapy highlights the need for additional study evaluating the mucosa over a longer period.

## Abbreviations

ECMO: Extra-corporeal membrane oxygenation
ARDS: Acute respiratory distress syndrome
I-FABP: V-V: Iintestinal - fatty acid binding protein
GAPDH: Glyceraldehyde-3-Phosphate Dehydrogenase
